# Decompressive craniectomy in subarachnoid hemorrhage compared to other etiologies: An institutional experience of 11 years

**DOI:** 10.1016/j.bas.2025.104203

**Published:** 2025-02-03

**Authors:** Emilia K. Pesonen, Aleksi Lammi, Cheng Qian, Mikael Von und Zu Fraunberg, Tommi K. Korhonen, Sami Tetri

**Affiliations:** Department of Neurosurgery, Oulu University Hospital & University of Oulu, Kajaanintie 52, 90029, Oulu, Finland

**Keywords:** Decompressive craniectomy, Subarachnoid hemorrhage, SAH, Stroke, Traumatic brain injury, GOSE

## Abstract

**Introduction:**

Decompressive craniectomy (DC) is a last-tier procedure to lower intracranial pressure in otherwise fatal brain injuries. DC significantly reduces mortality following traumatic brain injury (TBI) and ischemic stroke, but benefits in subarachnoid hemorrhage (SAH) are less clear.

**Research question:**

We compared the mortality and functional outcomes in patients who underwent DC after SAH with those who underwent DC following TBI or ischemic stroke.

**Materials and methods:**

All DC procedures performed in the Oulu University Hospital between January 2009 and December 2019 were retrospectively identified. Mortality and functional outcomes were assessed during a median follow-up of 20.7 months. Extended Glasgow Outcome Scale scores ≥5 were considered favorable.

**Results:**

One hundred twenty-four DCs were conducted to patients aged a median of 40 years (SD 16), of whom 88 (71%) were male. Fifty-eight (47%) DCs were due to TBI and 66 (53%) due to stroke. Of the strokes, 41 (62%) were ischemic and 21 (32%) SAH.

In multivariate models, the rates of unfavorable outcome were 48% in TBI, 78% in ischemic stroke (OR 2.73, 95% CI 0.70–10.64) and 86% in SAH (OR 3.15, 95%CI 0.67–14.77). Mortality rates were 22% in TBI, 17% in ischemic stroke (OR 0.50, 95%CI 0.11–2.31) and 33% in SAH (OR 0.97, 95%CI 0.24–3.99).

**Discussion and conclusion:**

Favorable outcomes were more common in TBI compared to stroke in univariate but not in multivariate analysis. There was no statistically significant difference in the rates of favorable outcomes between SAH and ischemic stroke.

## Highlights

• Seven of the 21 (33%) patients with SAH who underwent DC died, and 3/21 (14%) achieved a favorable outcome.
• There was no statistically significant difference in favorable outcome between SAH and ischemic stroke.
• Favorable functional outcome was more common in TBI compared to stroke in univariate but not in multivariate analysis.

## 1 Introduction

Decompressive craniectomy (DC) is a last-tier procedure to lower intracranial pressure (ICP) in patients with otherwise fatal brain injury. The surgery effectively controls ICP, affecting cerebral blood flow, oxygenation and metabolism (Timofeev et al., 2008), (Soustiel et al., 2010), (Schneider et al., 2002), (Lubillo et al., 2018). Consequently, survival improves following severe traumatic brain injury (TBI) and ischemic stroke (Vahedi et al., 2007), (Hutchinson et al., 2016). Extrapolating the above evidence, DC has been utilized to decrease ICP in conditions such as aneurysmal subarachnoid and intraparenchymal hemorrhage, sinus thrombosis, intracranial infections and metabolic encephalopathies (Takeuchi et al., 2013), (Nguyen et al., 2011), (Levi et al., 2021), (Alotaibi et al., 2017), (Aaron et al., 2013).

A meta-analysis found that DC is required in 10% of patients with aneurysmal subarachnoid hemorrhage (SAH), with younger age and concurrent ICH increasing the likelihood of DC (Darkwah Oppong et al., 2020). Elevated ICP is common after SAH and associated with worsened outcomes (Heuer et al., 2004), (Broderick et al., 1994). However, it is unclear whether this association reflects the impact of elevated ICP or other factors, such as the severity of SAH, on outcome (Heuer et al., 2004). The published results on the beneficial effects of DC following SAH have been conflicting. Although DC appears to effectively decrease ICP (Tuzgen et al., 2012), (Schirmer et al., 2007), this may not translate to a clinical outcome benefit (Alotaibi et al., 2017), (Nagel et al., 2009). This is despite improved cerebral perfusion and biomarkers of cerebral metabolism after DC (Nagel et al., 2009). Furthermore, a potential increase in survival might result from an increased number of severely disabled patients with a poor quality of life (Alotaibi et al., 2017).

We conducted an institutional review of all DCs conducted in our institution with the purpose of comparing mortality and functional outcomes after SAH to those after TBI and ischemic stroke.

## 2 Materials and methods

### 2.1 Data collection

We retrospectively identified all consecutive patients (n = 124) who had undergone a DC in the Oulu University Hospital between January 1, 2009 and December 31, 2019. Clinical data on demographics, including sex assigned at birth, previous medical history and the perioperative period, were collected. Pupillary status was collected at the latest available time before the DC. Functional outcomes were measured using the extended Glasgow Outcome Scale (GOSE) available from the patient records of the follow-up visit. Favorable recovery was defined as GOSE ≥5.

### 2.2 Decompressive craniectomy

In this study, DC was defined as a supratentorial procedure aiming to decrease ICP by removing a large part of the skull bone as described previously, and termed hemicraniectomy or bifrontal craniectomy depending on operative technique (Timofeev et al., 2012). A procedure was defined as a primary DC, if it was conducted immediately when the patient first arrived to the hospital, or if a DC was required during another surgery, such as aneurysm clipping or hematoma removal (Compagnone et al., 2005). Secondary DC was defined as a DC that was performed as the last line of treatment for elevated ICP. Elevated ICP was defined as ICP >20 mmHg for a minimum of 15 min despite maximal neurocritical care. The minimum DC size in all patients was 12 × 15cm according to an institutional protocol. Procedures, where a conventional pterional craniotomy bone flap was not replaced, were not included in the present series, unless a hemicraniectomy or bifrontal craniectomy was conducted in the same procedure. Oulu University Hospital is the only institution that performs DC in Northern Finland with a population of 741,162 inhabitants.

Patients with TBI were selected to DC using a stepwise treatment scheme (Hutchinson et al., 2016) consisting of routine neurointensive TBI care including sedation, intubation, ventilation, ICP monitoring, hypertonic fluids, and external ventricular drainage (EVD), applied as indicated by clinical circumstances (Hawryluk et al., 2019). Routine ICP monitoring was conducted in all TBI patients with a Glasgow Coma Scale (GCS) score ≤8, and EVD was placed if deemed beneficial on neurosurgical clinicoradiological assessment. A similar approach was applied for patients with SAH following the routine commencement of intravenous nimodipine and levetiracetam administration. DC was conducted when all other treatment options had been exhausted.

DC was performed as a last-line of treatment in patients with SAH aged ≤65 years, if the overall GCS score decreased a minimum of two points and ICP remained persistently above 20 mmHg. All patients with SAH and GCS ≤14 or significant hydrocephalus received an EVD and underwent routine ICP monitoring using the EVD. Intraparenchymal ICP probes were used additionally if EVD-based monitoring was deemed unreliable, such as in the presence of a large intraventricular hemorrhage. EVDs were placed emergently when a reduced GCS score was diagnosed.

DC was performed in ischemic stroke following a unilateral middle cerebral artery (MCA) or a carotid artery stroke if patients had a large hemispherical space-occupying infarction, were aged ≤65 years and the overall GCS score decreased a minimum of two or the motor response decreased a minimum of one point.

### 2.3 Statistical analysis

Statistical analysis was conducted using uni- and multivariate binary logistic regression analysis with favorable recovery (GOSE ≥5) and mortality during the follow-up period as the primary and secondary outcomes, respectively. Follow-up time was calculated as the time from DC to the latest available GOSE score in the patient records. The odds ratio (OR) and 95% confidence interval (CI) are reported. Mean values are reported with standard deviation (SD), and median values with interquartile range (IQR). Outcomes were compared between TBI and stroke (hemorrhagic stroke, including ICH and SAH, ischemic stroke and sinus thrombosis), and further between ischemic stroke and SAH. As a concurrent ICH is a well-established predictor of poor outcome in SAH (Lindner et al., 2023a), (Pegoli et al., 2015), (Rosengart et al., 2007), (Wan et al., 2016), patients with/without intracerebral hemorrhage were also analyzed separately. Statistical analyses were conducted using the SPSS (v. 23, IBM, Armonk, NY).

This study was approved by the medical director of the Oulu University Hospital and conducted in accordance with the Declaration of Helsinki. Research ethics committee review and patient consent were waived as only retrospective means of data collection were used, and the patients were not contacted.

## 3 Results

### 3.1 Demographic data

One hundred and twenty-four DCs had been conducted during the study period. The mean age of the patients was 40 years (SD 16, range 8–70), and 88 (71%) were male. Fifty-eight (47%) of the surgeries had been conducted due to TBI, and 66 (53%) due to stroke. Of the strokes, 41 (62%) were ischemic, 21 (32%) SAHs with (n = 10, 15%) or without (n = 11, 17%) an intracerebral hemorrhage (ICH) component, three (5%) spontaneous ICHs and one sinus thrombosis (2%).

Follow-up times, pupillary status and frequency of anticoagulant or antithrombotic use were similar between the SAH, TBI and ischemic stroke groups (Table 1). Patients with TBI were more likely to be male and younger compared to patients with SAH or ischemic stroke. Patients with ischemic stroke had higher pre-operative GCS scores than those with TBI or SAH (Table 1). Demographic data is reported in Table 2, Table 3.

**Table 1:** Subgroup characteristics of 120 patients with TBI, ischemic stroke and SAH who had undergone DC.

Characteristic	TBI (n = 58)	Ischemic stroke (n = 41)	SAH (n = 21)	P value
Sex, n (%)				**0.001**
Male	50 (86)	26 (63)	10 (48)	
Female	8 (14)	15 (37)	11 (52)	
Mean age (SD)	31 (58)	50 (40)	50 (21)	**<0.001**
DC type, n (%)				**0.01**
Primary	20 (34)	4 (10)	3 (14)	
Secondary	38 (66)	37 (90)	18 (86)	
Latest pupil status before DC, n (%)				0.67
Normal	35 (60)	28 (68)	14 (67)	
Asymmetry	20 (34)	13 (32)	7 (33)	
Symmetrically dilated	3 (5)	0 (0)	0 (0)	
Mean GCS on scene (SD)	7 (4.07)	12 (2.99)	8 (4.48)	0.20
Alcohol abuse				**0.02**
Yes	16 (28)	4 (10)	1 (5)	
No	42 (72)	37 (90)	20 (95)	
Anticoagulant or antithrombotic use, n (%)				0.28
Yes	4 (7)	7 (17)	2 (10)	
No	54 (93)	34 (83)	19 (90)	

Statistically significant comparisons in bold. Reference indicates reference category.

Statistical analyses conducted using the χ2, Fisher's exact, one-way analysis of variance and Brown-Forsythe tests as appropriate.

One patient with a cerebral venous sinus thrombosis, three patients with spontaneous, non-aneurysmal ICHs, four patients with tumors and two patients with infections were excluded from the analysis.

**ABBREVIATIONS** DC = decompressive craniectomy; GCS = Glasgow Coma Scale; ICH = intracerebral hemorrhage; SAH = subarachnoid hemorrhage; SD = standard deviation; TBI = traumatic brain injury.

**Table 2:** Functional outcomes of patients who had undergone DC (n = 124) based on univariate analysis. Favorable outcome was defined as GOSE score ≥5.

Characteristic	Favorable (n = 43) (Reference)	Unfavorable (n = 81)	OR (95% CI)
Sex, n (%)			
Male	31 (60)	57 (70)	Reference
Female	12 (40)	24 (30)	1.09 (0.48–2.47)
Mean age (SD)	30 (14)	45 (14)	**1.07 (1.04**–**1.10)**
DC type, n (%)			
Primary	9 (21)	18 (22)	Reference
Secondary	34 (79)	63 (78)	0.93 (0.38–2.28)
Primary diagnosis, n (%) a			
TBI	30 (71)	28 (36)	Reference
Ischemic stroke	9 (21)	32 (41)	**3.81 (1.55**–**9.38)**
SAH	3 (7)	18 (23)	**6.43 (1.71**–**24.22)**
Latest pupil status before DC, n (%)			
Normal	33 (77)	46 (57)	Reference
Asymmetry	9 (21)	33 (41)	**2.63 (1.11**–**6.23)**
Symmetrically dilated	1 (2)	2 (2)	1.44 (0.13**–**16.49)∗
Mean GCS on scene (SD)	9 (4)	9 (5)	1.01 (0.93–1.10)
Alcohol abuse			
Yes	6 (14)	15 (19)	1.40 (0.50–3.92)
No	37 (86)	66 (81)	Reference
Anticoagulant or antithrombotic use, n (%)			
Yes	2 (5)	11 (14)	3.22 (0.68–15.26)
No	41 (95)	70 (86)	Reference
Median follow-up time, months (IQR)	27 (10–51)	12 (1–53)	1.00 (0.98–1.01)

Favorable outcome was chosen as the reference category. Reference indicates reference category. Statistically significant comparisons in bold.

**ABBREVIATIONS** CI = confidence interval; DC = decompressive craniectomy; GCS = Glasgow Coma Scale; GOSE = Glasgow Outcome Scale Extended; ICH = intracerebral hemorrhage; IQR = interquartile range; OR = odds ratio; SAH = subarachnoid hemorrhage; SD = standard deviation; TBI = traumatic brain injury.

a One patient with a cerebral sinus thrombosis and three patients with spontaneous, non-aneurysmal ICHs were excluded from the analysis.

**Table 3:** The overall mortality of patients who had undergone DC (n = 124) based on univariate analysis.

Characteristic	Dead (n = 27)	Alive (n = 97) (Reference)	OR (95% CI)
Sex, n (%)			
Male	22 (81)	66 (65)	Reference
Female	5 (19)	31 (30)	0.48 (0.17–1.40)
Mean age (SD)	48 (15)	38 (16)	**1.05 (1.01**–**1.08)**
DC type, n (%)			
Primary	7 (26)	20 (21)	Reference
Secondary	20 (74)	77 (79)	0.74 (0.28–2.00)
Primary diagnosis, n (%) b			
TBI	13 (48)	45 (48)	Reference
Ischemic stroke	7 (26)	34 (37)	0.71 (0.26–1.98)
SAH	7 (26)	14 (15)	1.73 (0.58–5.19)
Latest pupil status before DC, n (%)			
Normal	9 (33)	70 (72)	Reference
Asymmetry	16 (59)	26 (27)	**4.79 (1.88**–**12.16)**
Symmetrically dilated	2 (7)	1 (1)	**15.56 (1.28**–**189.27)**^a^
Mean GCS on scene (SD)	7 (5)	10 (4)	**0.90 (0.82**–**0.99)**
Alcohol abuse			
Yes	7 (26)	14 (14)	2.08 (0.74–5.81)
	20 (74)	83 (86)	Reference
Anticoagulant or antithrombotic use, n (%)			
Yes	5 (19)	8 (8)	2.53 (0.75–8.49)
No	22 (81)	89 (92)	Reference
Median follow-up time, months (IQR)	0.3 (0.1–1.7)	27 (9.3–60.3)	0.90 (0.84–0.96)

Being alive was chosen as the reference category. Reference indicates reference category. Statistically significant comparisons in bold.

**ABBREVIATIONS** CI = confidence interval; DC = decompressive craniectomy; GCS = Glasgow Coma Scale; GOSE = Glasgow Outcome Scale Extended; ICH = intracerebral hemorrhage; IQR = interquartile range; OR = odds ratio; SAH = subarachnoid hemorrhage; SD = standard deviation; TBI = traumatic brain injury.

a One patient with both pupils dilated survived with favorable outcome.

b One patient with a cerebral sinus thrombosis and three patients with spontaneous, non-aneurysmal ICHs were excluded from the analysis.

### 3.2 Outcomes

**Fig. 1:**
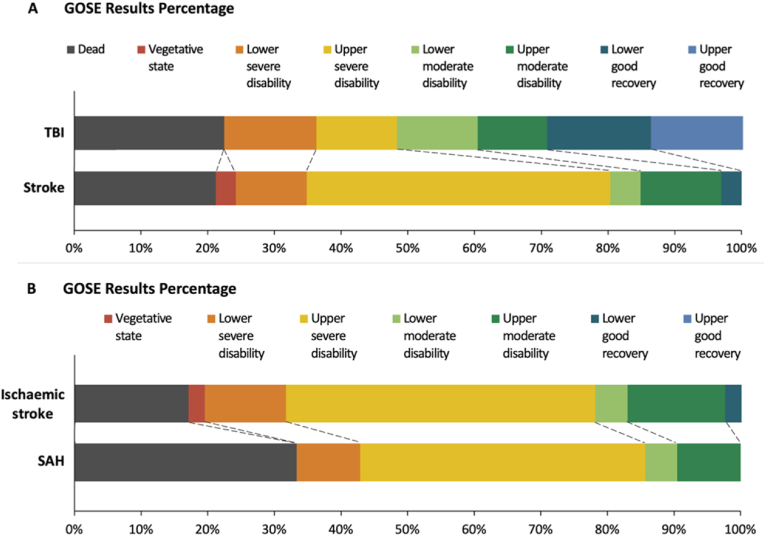
Stacked bar chart of the extended Glasgow Outcome Scale (GOSE) scores at the end of the follow-up period in patients who underwent decompressive craniectomy. **Upper**: The GOSE scores of patients with traumatic brain injury (TBI) and stroke. **Lower**: The GOSE scores in ischemic stroke and subarachnoid hemorrhage (SAH).

The mean GOSE of all patients was 4.0 (SD 2.1) after median follow-up time of 21 months (IQR 5–52), and GOSE among the survivors was 4.9 (SD 1.6) after 27 months (IQR 9–60) (Fig. 1). Patients after TBI had achieved favorable outcome more commonly than after stroke (Table 2 & Fig. 1), but rates of favorable outcome did not differ statistically between SAH and ischemic stroke (Table 4). Of the 11 patients with SAH and a concurrent ICH, none (0%) had a favorable outcome, whereas 3/10 (30%) patients without a concurrent ICH had a favorable outcome (Fisher's exact p = 0.09). The overall mortality rate was 22% (n = 27), with 22% (n = 13) mortality after TBI and 22% (n = 14) after SAH or ischemic stroke combined.

**Table 4:** Outcomes of the 62 patients with ischemic stroke or SAH.

Etiology^a^	Ischemic stroke (n = 41)	SAH (n = 21)	p-value
Favorable outcome	9 (22)	3 (14)	0.74
Unfavorable outcome	32 (78)	18 (86)	
Dead	7 (17)	7 (33)	0.20

Favorable outcome was defined as GOSE score ≥5 and unfavorable outcome as GOSE score <5. Analyses conducted using the χ2 test.

**ABBREVIATIONS** GOSE = extended Glasgow Outcome Scale; SAH = subarachnoid hemorrhage.

a Mean follow-up times 35 (SD 35) and 33 (SD 30) months for ischemic stroke and SAH, respectively (p = 0.81).

### 3.3 Outcome predictors

In univariate analyses, older age, pupillary asymmetry, and stroke (hemorrhagic stroke, including ICH and SAH, ischemic stroke and sinus thrombosis) as the primary diagnosis were associated with an unfavorable outcome following DC, whereas sex, GCS on scene, alcohol abuse, and anticoagulant or antithrombotic use were not (Table 2). Older age, pupillary asymmetry and lower GCS on scene were associated with increased mortality, whereas sex, alcohol abuse, anticoagulant or antithrombotic use, and the primary diagnosis were not (Table 3).

In the multivariate models, patients with SAH or ischemic stroke as the primary diagnosis had similar rates of favorable outcome and mortality adjusted with primary diagnosis, pupil status and GCS (Table 5). Older age was associated with increased mortality and unfavorable outcome in the multivariate models. Pupillary asymmetry was associated with increased mortality, but not with functional outcome (Table 5).

**Table 5:** Predictors of unfavorable outcome and mortality in 124 DC patients obtained by multivariate analysis.

Variable	Unfavorable outcome	Mortality
	OR (95% CI)	OR (95% CI)
Age at DC	**1.07 (1.03**–**1.11)**	**1.09 (1.04**–**1.14)**
Primary diagnosis		
TBI	Reference	Reference
Ischemic stroke	2.73 (0.70–10.64)	0.50 (0.11–2.31)
SAH	3.15 (0.67–14.77)	0.97 (0.24–3.99)
Latest pupil status before DC		
Normal	Reference	Reference
Asymmetry	**2.88 (1.02**–**8.09)**	**7.16 (2.34**–**21.88)**
Symmetrically dilated^a^	6.76 (0.44–104.34)	**112.21 (4.57**–**2753.90)**^a^
GCS on scene	0.90 (0.80–1.02)	0.90 (0.79–1.04)

Favorable outcome was defined as GOSE score ≥5 and unfavorable outcome as GOSE score <5. Reference indicates reference category. Statistically significant comparisons in bold.

**ABBREVIATIONS** CI = confidence interval; DC = decompressive craniectomy; GCS = Glasgow Coma Scale; GOSE = Glasgow Outcome Scale Extended; ICH = intracerebral hemorrhage; OR = odds ratio; SAH = subarachnoid hemorrhage; TBI = traumatic brain injury.

a One patient with both pupils dilated survived with favorable outcome.

Overall mortality was statistically similar between SAH patients with or without ICH (Table 6). However, 0/11 (0%) of the patients with SAH and had a favorable outcome, whereas 3/10 (30%) of the patients with SAH in the absence of ICH had a favorable outcome (Table 6).

**Table 6:** The outcomes of SAH patients with and without ICH.

	SAH with ICH (n = 11)	SAH only (n = 10)	OR (95% CI)
		Reference	
Functional outcome			N/A^a^
Favorable	0 (0)	3 (30)	
Unfavorable	11 (100)	7 (70)	
Mortality			1.33 (0.21–8.29)
Dead	4 (36)	3 (30)	
Alive	7 (64)	7 (70)	Reference

Favorable outcome was defined as GOSE score ≥5 and unfavorable outcome as GOSE score <5.

**ABBREVIATIONS** CI = confidence interval; DC = decompressive craniectomy; ICH = intracerebral hemorrhage; OR = odds ratio; SAH = subarachnoid hemorrhage.

a 0 favorable outcomes in the SAH with ICH group.

## 4 Discussion

### 4.1 Decompressive craniectomy following subarachnoid hemorrhage

The evidence base for DC in TBI and ischemic stroke is robust in terms of mortality reduction (Vahedi et al., 2007), (Hutchinson et al., 2016), but the decrease in mortality rates may result may result in increased rates of unfavorable outcomes (Otani and Yoshino, 2021), (Lemcke et al., 2010). However, functional status may improve several years after DC (Kolias et al., 2022), (Geurts et al., 2013). Since elevated ICP increases mortality after SAH (Magni et al., 2015), DC may improve functional outcomes following poor-grade SAH (Brandecker et al., 2021). In our comparative analysis, although patients with ischemic stroke had higher pre-operative GCS scores than those with SAH, the outcomes were similar for both groups.

At our institution, the mortality rate for patients with DC following SAH was 33%. A recent cohort study reported 6-month mortality rates of 22–40% in patients with SAH after DC (Güresir et al., 2009). Fourteen percent attained GOSE ≤5 in our SAH cohort, but higher rates (27%) of favorable outcome have been reported (Güresir et al., 2009). This difference may be due to different outcome measures as the latter study used a modified Rankin scale score of 0–3 in defining a favorable outcome.

The clinical outcome benefit of DC following poor-grade SAH remains unclear (Alotaibi et al., 2017), and some institutions do not offer DC in the context of SAH despite intracranial hypertension. None of the SAH patients with a concurrent ICH had a favorable outcome in our cohort, whereas 30% of SAH patients with no concurrent ICH had a favorable outcome. Indeed, a concurrent ICH is a well-established predictor of poor outcome in SAH (Lindner et al., 2023a), (Pegoli et al., 2015), (Rosengart et al., 2007), (Wan et al., 2016). As expected, functional outcomes in SAH were better in younger patients (Lindner et al., 2023b), (Wilson et al., 2013). This is consistent with potentially superior long-term recovery capacity compared to more elderly patients. Long-term functional improvements have been reported in up to 73% of SAH survivors (Lindner et al., 2023b), (Wilson et al., 2013).

### 4.2 Outcomes in other etiologies

Patients with ischemic stroke or SAH had poorer functional outcomes compared to TBI in univariate but not in multivariate analysis. This difference was likely due to the older age of patients diagnosed with stroke compared to TBI (at mean 50 vs. 31 years, respectively). Reflecting the best-established indications for DC, TBI was the most common primary diagnosis leading to DC (48%), followed by ischemic stroke (34%), (Vahedi et al., 2007), (Hutchinson et al., 2016).

In our study, the mortality rate of patients with TBI was 22% after a median follow-up period of 14 months, which is in line with the 6-month post-DC mortality rate of 21% in a Swedish retrospective study of 61 patients with TBI (Svedung Wettervik et al., 2021). In comparison, the 24-month mortality rate for patients with traumatic intracranial hypertension after DC was 34% in the RESCUEicp long-term follow-up (Kolias et al., 2022). Fifty-two percent of patients with TBI had a favorable outcome in our cohort compared to 36% in Kolias et al. (2022). A prospective non-randomized control study reported 49% favorable outcomes (Decraene et al., 2023). Differences in clinical practice compared to strict protocols of a randomized research and differing cohort demographics might explain most of the variation in results.

The 17% mortality rate in our ischemic stroke group following DC is consistent with previous research (Vahedi et al., 2007), (Jüttler et al., 2007). Twenty-two percent of patients with ischemic stroke had a favorable outcome, Since the modified Rankin Scale is commonly used in measuring outcomes in stroke, direct comparison of outcomes to previous research is challenging (Van Swieten et al., 1988), (Farrell et al., 1991).

### 4.3 Clinical variables and craniectomy outcome

As expected, older age predicted worse functional outcomes following DC (Soleman et al., 2021). Preoperative anisocoria was associated with worse outcomes in the univariate model, but not in the multivariate model. This is due to one 16-year-old with TBI who had a favorable outcome despite preoperatively bilaterally fixed and dilated pupils. His good outcome is probably explained by the short delay to DC as his pupil status worsened in the operating theatre whilst being prepared for surgery.

Interestingly, the initial GCS score was associated with mortality but not with functional outcomes. Even though several studies have found that initial GCS score is a strong predictor of outcome after DC (Shen et al., 2024), (Raffiq et al., 2014), (Ucar et al., 2005), (Rafieezadeh et al., 2024), initial GCS might not be an optimal predictor of longer-term functional outcomes in TBI (Zafonte et al., 1996). We hypothesize that our results reflect the large geographical area served by our institution leading to considerable variation in the transportation times from the scene to the hospital. The long delays could result in significant deterioration of the patient's clinical condition before arriving to the hospital. Consequently, the GCS on scene might not accurately reflect the clinical status of all patients on arrival to the hospital.

The evidence on cost-effectiveness of DC in etiologies other than SAH is mixed (Hofmeijer et al., 2013), (Malmivaara et al., 2011a), (Malmivaara et al., 2011b), (Bhattacharyya et al., 2019). One explanation is that recovery can occur over several years. Studies using shorter time intervals might, therefore, show reduced mortality with limited improvements in favorable outcomes (Behranwala et al., 2021). A study analyzing the cost-effectiveness of DC in SAH would be valuable in determining the role of DC in SAH. A randomized study comparing DC to the current standard treatment in SAH is currently ongoing (Güresir et al., 2022). Supplemental to RCTs, multi-center registries could provide valuable real-world data on outcome predictors following DC in SAH helping to optimize patient selection.

### 4.4 Limitations

This study should be interpreted in light of its retrospective nature as patient selection to DC was based on individual clinical assessment in addition to clinical protocols. As such, an element of selection bias is inherent in this observational work. Another limitation is the non-standardized outcome collection times, although the median follow-up time (27 months) is likely sufficient to estimate long-term outcomes, as the active rehabilitation periods are complete. The proportions of primary diagnoses in our cohort mirror the overall incidence of the diseases, but the small size of some subgroups may increase the effect of chance on the outcomes, especially in SAH.

No universal measure of functional outcome currently exists. The authors chose the GOSE because it is frequently used in DC research, although originally developed for TBI (Jennett and Bond, 1975). This may bias outcome evaluation after stroke, in which mRS is the more established outcome measure. However, the categories somewhat overlap and likely correlate with one another (Van Swieten et al., 1988), (Farrell et al., 1991), (Gaastra et al., 2022).

Treatment limiting decisions are a common consideration in patients requiring DC, and may influence the observed survival and functional outcomes. Indeed, based on a previous propensity score analysis, a substantial proportion of patients with TBI whose life-sustaining treatment was withdrawn may have been able to survive (Sanders et al., 2024). However, the process of making treatment limiting decisions was relatively consistent in the present single-center study.

## 5 Conclusion

After DC, patients with TBI had more favorable outcomes at 3–6 months compared to the stroke group (hemorrhagic stroke, including ICH and SAH, ischemic stroke and sinus thrombosis) in univariate but not in multivariate analysis. There was no significant difference in functional outcomes between SAH and ischemic stroke.

## Data statement

Anonymized data will be made available upon reasonable request to the corresponding author (subject to the Finnish regulations).

## Declaration of competing interest

The authors declare that they have no known competing financial interests or personal relationships that could have appeared to influence the work reported in this paper.

## References

[bib1] Aaron S. (2013). Decompressive craniectomy in cerebral venous thrombosis: a single centre experience. J. Neurol. Neurosurg. Psychiatry.

[bib2] Alotaibi N.M. (2017). Effects of decompressive craniectomy on functional outcomes and death in poor-grade aneurysmal subarachnoid hemorrhage: a systematic review and meta-analysis. J. Neurosurg..

[bib3] Behranwala R., Aojula N., Hagana A., Houbby N., de Preux D.L. (2021). An economic evaluation for the use of decompressive craniectomy in the treatment of refractory traumatic intracranial hypertension. Brain Inj..

[bib4] Bhattacharyya A., Tahir A., Chandrashekar A., Vasisht S., Stinson L., Omatseye J. (2019). A cost-utility analysis of decompressive hemicraniectomy versus medical treatment in the management of space-occupying brain oedema post middle cerebral artery infarction. Eur. J. Neurol..

[bib5] Brandecker S., Hadjiathanasiou A., Kern T., Schuss P., Vatter H., Güresir E. (2021). Primary decompressive craniectomy in poor-grade aneurysmal subarachnoid hemorrhage: long-term outcome in a single-center study and systematic review of literature. Neurosurg. Rev..

[bib6] Broderick J.P., Brott T.G., Duldner J.E., Tomsick T., Leach A. (1994). Initial and recurrent bleeding are the major causes of death following subarachnoid hemorrhage. Stroke.

[bib7] Compagnone C. (2005). The management of patients with intradural post-traumatic mass lesions: a multicenter survey of current approaches to surgical management in 729 patients coordinated by the European Brain Injury Consortium. Neurosurgery.

[bib8] Darkwah Oppong M., Golubovic J., Hauck E.F., Wrede K.H., Sure U., Jabbarli R. (2020). Decompressive craniectomy in aneurysmal subarachnoid hemorrhage: who and when? – a systematic review and meta-analysis. Clin. Neurol. Neurosurg..

[bib9] Decraene B. (2023). Decompressive craniectomy as a second/third-tier intervention in traumatic brain injury: a multicenter observational study. Injury.

[bib10] Farrell B., Godwin J., Richards S., Warlow C. (1991). The United Kingdom transient ischaemic attack (UK-TIA) aspirin trial: final results. J. Neurol. Neurosurg. Psychiatry.

[bib11] Gaastra B. (2022). Evidence-based interconversion of the Glasgow Outcome and modified Rankin scales: pitfalls and best practices. J. Stroke Cerebrovasc. Dis..

[bib12] Geurts M., Van Der Worp H.B., Kappelle L.J., Amelink G.J., Algra A., Hofmeijer J. (2013). Surgical decompression for space-occupying cerebral infarction: outcomes at 3 years in the randomized HAMLET trial. Stroke.

[bib13] Güresir E., Schuss P., Vatter H., Raabe A., Seifert V., Beck J. (2009). Decompressive craniectomy in subarachnoid hemorrhage. Neurosurg. Focus.

[bib14] Güresir E. (2022). PrImary decompressive Craniectomy in AneurySmal Subarachnoid hemOrrhage (PICASSO) trial: study protocol for a randomized controlled trial. Trials.

[bib15] Hawryluk G.W.J. (2019). A management algorithm for patients with intracranial pressure monitoring: the Seattle international severe traumatic brain injury consensus conference (SIBICC). Intensive Care Med..

[bib16] Heuer G.G., Smith M.J., Elliott J.P., Winn H.R., Leroux P.D. (2004). Relationship between intracranial pressure and other clinical variables in patients with aneurysmal subarachnoid hemorrhage. J. Neurosurg..

[bib17] Hofmeijer J., Van Der Worp H.B., Kappelle L.J., Eshuis S., Algra A., Greving J.P. (2013). Cost-effectiveness of surgical decompression for space- occupying hemispheric infarction. Stroke.

[bib18] Hutchinson P.J. (2016). Trial of decompressive craniectomy for traumatic intracranial hypertension. N. Engl. J. Med..

[bib19] Jennett B., Bond M. (1975). Assessment of outcome after severe brain damage. Lancet.

[bib20] Jüttler E. (2007). Decompressive surgery for the treatment of malignant infarction of the middle cerebral artery (DESTINY): a randomized, controlled trial. Stroke.

[bib21] Kolias A.G. (2022). Evaluation of outcomes among patients with traumatic intracranial hypertension treated with decompressive craniectomy vs standard medical care at 24 Months: a secondary analysis of the RESCUEicp randomized clinical trial. JAMA Neurol..

[bib22] Lemcke J., Ahmadi S., Meier U. (2010). Outcome of patients with severe head injury after decompressive craniectomy. Acta Neurochir. Suppl..

[bib23] Levi N., Baker H., Ben-Chetrit E., Levine P., Margalit N., Winestone J. (2021). Decompressive craniectomy for treatment of elevated intracranial pressure in community-acquired bacterial meningitis: case study, literature review, and proposed guidelines. Interdiscipl Neurosurg..

[bib24] Lindner A. (2023). The location of intraparenchymal bleeding determines functional outcome after spontaneous subarachnoid hemorrhage. Eur. J. Neurol..

[bib25] Lindner A. (2023). Long-term clinical trajectory of patients with subarachnoid hemorrhage: linking acute care and neurorehabilitation. Neurocrit Care.

[bib26] Lubillo S.T. (2018). Prognostic value of changes in brain tissue oxygen pressure before and after decompressive craniectomy following severe traumatic brain injury. J. Neurosurg..

[bib27] Magni F., Pozzi M., Rota M., Vargiolu A., Citerio G. (2015). High-resolution intracranial pressure burden and outcome in subarachnoid hemorrhage. Stroke.

[bib28] Malmivaara K., Ohman J., Kivisaari R., Hernesniemi J., Siironen J. (2011). Cost-effectiveness of decompressive craniectomy in non-traumatic neurological emergencies. Eur. J. Neurol..

[bib29] Malmivaara K., Kivisaari R., Hernesniemi J., Siironen J. (2011). Cost-effectiveness of decompressive craniectomy in traumatic brain injuries. Eur. J. Neurol..

[bib30] Nagel A., Graetz D., Vajkoczy P., Sarrafzadeh A.S. (2009). Decompressive craniectomy in aneurysmal subarachnoid hemorrhage: relation to cerebral perfusion pressure and metabolism. Neurocrit Care.

[bib31] Nguyen H.S., Callahan J.D., Cohen-Gadol A.A. (2011). Life-saving decompressive craniectomy for diffuse cerebral edema during an episode of new-onset diabetic ketoacidosis: case report and review of the literature. Childs Nerv Syst.

[bib32] Otani N., Yoshino A. (2021). [Decompressive craniectomy for intracranial hypertension]. Noshinkeigeka.

[bib33] Pegoli M., Mandrekar J., Rabinstein A.A., Lanzino G. (2015). Predictors of excellent functional outcome in aneurysmal subarachnoid hemorrhage. J. Neurosurg..

[bib34] Raffiq M.A.M., Haspani M.S.M., Kandasamy R., Abdullah J.M. (2014). Decompressive craniectomy for malignant middle cerebral artery infarction: impact on mortality and functional outcome. Surg. Neurol. Int..

[bib35] Rafieezadeh A., Prabhakaran K., Sadeghian A., Jose A.M., Zangbar B. (2024). Optimizing traumatic brain injury recovery: exploring the advantages and results of decompressive craniectomy. J. Assoc. Phys. India.

[bib36] Rosengart A.J., Schultheiss K.E., Tolentino J., Macdonald R.L. (2007). Prognostic factors for outcome in patients with aneurysmal subarachnoid hemorrhage. Stroke.

[bib37] Sanders W.R. (2024). Recovery potential in patients who died after withdrawal of life-sustaining treatment: a TRACK-TBI propensity score analysis. J. Neurotrauma.

[bib38] Schirmer C.M., Hoit D.A., Malek A.M. (2007). Decompressive hemicraniectomy for the treatment of intractable intracranial hypertension after aneurysmal subarachnoid hemorrhage. Stroke.

[bib39] Schneider G.H., Bardt T., Lanksch W.R., Unterberg A. (2002). Decompressive craniectomy following traumatic brain injury: ICP, CPP and neurological outcome. Acta Neurochir. Suppl..

[bib40] Shen J. (2024). Factors associated with mortality and functional outcome after decompressive craniectomy in malignant middle cerebral artery infarction. BMC Neurol..

[bib41] Soleman J., Ullmann M., Greuter L., Ebel F., Guzman R. (2021). Mortality and outcome in elderly patients undergoing emergent or elective cranial surgery. World Neurosurg.

[bib42] Soustiel J.F., Sviri G.E., Mahamid E., Shik V., Abeshaus S., Zaaroor M. (2010). Cerebral blood flow and metabolism following decompressive craniectomy for control of increased intracranial pressure. Neurosurgery.

[bib43] Svedung Wettervik T., Lenell S., Enblad P., Lewén A. (2021). Decompressive craniectomy in traumatic brain injury-craniectomy-related and cranioplasty-related complications in a single center. World Neurosurg.

[bib44] Takeuchi S., Wada K., Nagatani K., Otani N., Mori K. (2013). Decompressive hemicraniectomy for spontaneous intracerebral hemorrhage. Neurosurg. Focus.

[bib45] Timofeev I. (2008). Effect of decompressive craniectomy on intracranial pressure and cerebrospinal compensation following traumatic brain injury. J. Neurosurg..

[bib46] Timofeev I., Santarius T., Kolias A.G., Hutchinson P.J.A. (2012). Decompressive craniectomy - operative technique and perioperative care. Adv. Tech. Stand. Neurosurg..

[bib47] Tuzgen S., Kucukyuruk B., Aydin S., Ozlen F., Kizilkilic O., Abuzayed B. (2012). Decompressive craniectomy in patients with cerebral infarction due to malignant vasospasm after aneurysmal subarachnoid hemorrhage. J. Neurosci. Rural Pract..

[bib48] Ucar T., Akyuz M., Kazan S., Tuncer R. (2005). Role of decompressive surgery in the management of severe head injuries: prognostic factors and patient selection. J. Neurotrauma.

[bib49] Vahedi K. (2007). Early decompressive surgery in malignant infarction of the middle cerebral artery: a pooled analysis of three randomised controlled trials. Lancet Neurol..

[bib50] Van Swieten J.C., Koudstaal P.J., Visser M.C., Schouten H., Van Gijn J. (1988). Interobserver agreement for the assessment of handicap in stroke patients. Stroke.

[bib51] Wan A., Jaja B.N.R., Schweizer T.A., Macdonald R.L. (2016). Clinical characteristics and outcome of aneurysmal subarachnoid hemorrhage with intracerebral hematoma. J. Neurosurg..

[bib52] Wilson D.A., Nakaji P., Albuquerque F.C., McDougall C.G., Zabramski J.M., Spetzler R.F. (2013). Time course of recovery following poor-grade SAH: the incidence of delayed improvement and implications for SAH outcome study design. J. Neurosurg..

[bib53] Zafonte R.D., Hammond F.M., Mann N.R., Wood D.L., Black K.L., Millis S.R. (1996). Relationship between Glasgow coma scale and functional outcome. Am. J. Phys. Med. Rehabil..

