# Supplementary material for: Decompressive craniectomy in subarachnoid hemorrhage compared to other etiologies: An institutional experience of 11 years
Source: Brain Spine. 2025 Feb 3;5:104203. doi: 10.1016/j.bas.2025.104203 (PMC11850783; doi:10.1016/j.bas.2025.104203)
Supplement: Multimedia component 1 [file mmc1.docx]

| **SUPPLEMENTAL TABLE 1. Subgroup characteristics of patients with SAH who had undergone DC.** | | | |
| --- | --- | --- | --- |
| Characteristic | SAH with ICH (n = 11) | SAH only (n = 10) | P value |
| Sex, n (%) |  |  | 0.59 |
| Male | 5 (45) | 5 (50) |  |
| Female | 6 (55) | 5 (50) |  |
| Mean age (SD) | 51 (6) | 48 (13) | 0.50 |
| DC type, n (%) |  |  | 0.44 |
| Primary | 3 (27) | 4 (40) |  |
| Secondary | 8 (73) | 6 (60) |  |
| Latest pupil status before DC, n (%) |  |  | .22 |
| Normal | 6 (55) | 8 (80) |  |
| Asymmetry | 5 (45) | 2 (20) |  |
| Mean GCS on scene (SD) | 8.0 (3.8) | 8.8 (5.3) | 0.69 |
| Alcohol abuse |  |  | 0.52 |
| Yes | 1 (9) | 0 (0) |  |
| No | 10 (91) | 10 (100) |  |
| Anticoagulant or antithrombotic use, n (%) |  |  | 0.26 |
| Yes | 2 (18) | 0 (0) |  |
| No | 9 (82) | 10 (100) |  |

Reference indicates reference category. Statistically significant comparisons in bold.

Statistical analyses conducted using the χ2, Fisher’s exact, one-way analysis of variance and Brown-Forsythe tests as appropriate.

**ABBREVIATIONS** DC = decompressive craniectomy; GCS = Glasgow Coma Scale; ICH = intracerebral hemorrhage; SAH = subarachnoid hemorrhage; SD = standard deviation.
